# Trends and correlates of intimate partner violence (IPV) victimization in Rwanda: results from the 2015 and 2020 Rwanda Demographic Health Survey (RDHS 2015 and 2020)

**DOI:** 10.1186/s12905-022-01951-3

**Published:** 2022-09-06

**Authors:** Claire Bahati, Josias Izabayo, Pascaline Munezero, Japhet Niyonsenga, Léon Mutesa

**Affiliations:** 1 Department of Clinical Psychology, College of Medicine and Health Sciences, University of Rwanda, Kigali, Rwanda; 2 Centre for Mental Health, College of Medicine and Health Sciences, University of Rwanda, Kigali, Rwanda; 3 Mental Health and Behavioral Research Group, College of Medicine and Health Sciences, University of Rwanda, Kigali, Rwanda; 4 Centre of Human Genetics, College of Medicine and Health Sciences, University of Rwanda, Kigali, Rwanda

**Keywords:** Violence victimization, Intimate partner violence, DHS, Rwanda

## Abstract

**Background:**

Intimate partner violence (IPV) is reported to be a public health issue given its magnitude and long-lasting consequences. Men are generally thought to be perpetrators of IPV, but they can also be victims. In Rwanda, the experience of men as victims has not yet been described and characterized. The aim of this study is to examine the trends and correlates of IPV victimization for men and women in Rwanda.

**Methods:**

The data for this study were extracted from the Rwanda Demographic and Health Survey (RDHS) in 2014/15 (female: n = 8292, male: n = 3470) and 2019/2020 (female = 8574, male: n = 3590). The survey had used a structured measure of IPV (i.e. physical, sexual, or emotional) and its related demographic characteristics to collect data in a nationally representative sample of ever-married women aged 15–49 years and men aged 15–59 years. Multiple logistic regression was applied to examine the association between demographic characteristics and IPV in both women and men.

**Result:**

The prevalence of IPV among women increased from 40% in 2015 to 46% in 2020, while it decreased from 21 to 18% in men during the same time period. The associated factors for women IPV victimization in 2015 were: uneducated husband (Adjusted Odds Ratios (AOR) = 5.570, 95% CI 1.29–24.02), woman from the poorest household (AOR = 2.834, 95% CI 1.9–93.12), husband aged from 30 to 39 years (AOR = 2.797, 95% CI 1.517–5.158), husband consuming alcohol (AOR = 3.021, 95% CI 1.517–5.158); women involved in decisions about their own earnings (AOR = 0.576, 95% CI 0.37–0.88); and purchases (AOR = 0.472, 95% CI 0.27–0.82). However, the factors such as uneducated husbands (AOR = 3.032, 95% CI 1.117–8.24); husbands consuming alcohol (AOR = 1.712, 95% CI 2.408–4.486); a woman's involvement in decisions on her personal health (AOR = 0.443, 95% CI 0.30–0.63) and visits from her family or relatives (AOR = 0.405, 95% = 0.41–0.22) were factors of IPV in 2020. On the other hand, the associated factors for men IPV victimization in 2015 were being from richer wealth index (AOR = 0.21, 95% CI 0.04–1.04), frequency of being hit in last 12 months by other than partner (AOR = 5.49, 95% CI 1.65–18.25), woman often consuming alcohol (AOR = 13.30, 95% CI 1.9–93.12); whereas its associated factor in 2020 were women consuming alcohol (3.91, 95% CI 0.55–9.87).

**Conclusion:**

The present study revealed a significant increase in IPV against women, and slight decrease of IPV against men in Rwanda from 2015 to 2020, as well as its associated risks and protective factors over time. This increase needs further exploration given that government and partners have invested in policies and strategies to mitigate the IPV with limited impact. Since there is a relationship between IPV prevalence and education, the existing laws on domestic violence need to be known by the citizens. Findings from this study evidenced also visits from extended families to be a protective factor and therefore suggesting the necessity of a family and community-based approach in managing IPV in Rwanda. Future studies to assess the effectiveness of community-based approach in preventing IPV.

## Background

Intimate partner violence (IPV) refers to violence between two individuals involved in an intimate relationship, and it exists in all countries, cultures, and societies [1]. The World Health Organization defines IPV as “a behavior within an intimate relationship that causes physical, sexual, or psychological harm, including acts of physical aggression, sexual coercion, and psychological abuse and controlling behaviors” [2]. Intimate partner violence is considered as a public health and human rights issue. Prior research has shown that women who experienced physical or sexual intimate partner violence have a risk of developing physical problems [3–5], including difficulty accessing and using antenatal care services for pregnant women [6], as well as mental health issues such as depression, anxiety, posttraumatic stress disorder, suicide, and alcohol abuse [4].

Male victims of IPV violence have been severely neglected in public policy, but they are not uncommon; they can also be victims, but they are less likely to report it [7]. As revealed in prior studies [8–11], the reasons for men’s reluctance to report IPV in Rwandan context may include “refusal to view their experiences as abuse, hesitancy to identify with victimizing language, lack of available supportive services, embarrassment, shame, loss of masculinity, fear of being judged or disbelieved by others, fear of police response, and devotion to their family”. Also, it is thought that men experience more verbal abuse than physical abuse. Physical abuse can be reported to a third party especially if it results in injury, but verbal or psychological abuse may have nothing physical to show as evidence for it [11]. Although most of the prior studies focused mostly on women as victims, a review of 91 studies also showed that one in five men was also a victim of intimate partner violence [12–15] and that men who experienced IPV were likely to develop poor health outcomes. According to the World Health Organization’s estimates [16], 1 in 3 women have experienced physical or sexual violence or both of these forms of violence at least once in their lifetime globally.

Regionally, the prevalence of sexual or physical intimate partner violence was found to be higher in South East Asia (38%) and Africa (37%), compared to other regions such as America (30%) and Europe (25%) [17]. A similar study by the World Health Organization combining data from 161 countries ranked 19 countries with the highest prevalence of physical or sexual intimate partner violence among women aged 15–49 years. Sexual or physical IPV prevalence ranged from 53% in Kiribati and 52% in Figi to 40% in Burundi, Lesotho and Samoa. Rwanda is among the sixteen countries with the second highest prevalence range, with 35–39% of ever-married/partner aged 15–49 years experiencing physical or sexual violence [17]. There are numerous theories regarding the causes and consequences of IPV against women, particularly in Africa. The socio-ecological model categorizes the risk factors that influence the occurrence of IPV as individual, relationship, community, and societal level factors [18]. A young age, low level of education, unemployment, harmful alcohol use, witnessing parental violence, relationship quality, having multiple partners, poverty, culture, posttraumatic stress disorder, and social norms are among these factors [5, 18–20].

Rwanda continues to be one of the countries with the highest prevalence of intimate partner violence against women worldwide. Compared to other countries in the region, Rwanda has laws and legislation to protect women against violence. For example, in 2008, the Rwandan government implemented the Prevention and Punishment of Gender-Based Violence Law, which covers all forms of violence and includes a minimum prison sentence of six months [21]. According to Article 16 of law N°59/2008 of 10/09/2008 on prevention and punishment of Gender-Based Violence in Rwanda, sexual abuse or rape that results in terminal illness or death can result in life imprisonment. The Rwandan government has supported the establishment of various initiatives to combat gender-based violence (GBV) and IPV, such as prevention clubs in high schools and universities, village-level prevention committees, parents' evening forums to raise awareness, identify, and assist victims of violence, and GBV desks at the Ministry of Defense and National Police. In 2009, the Rwanda National Police and the Ministry of Health also established One-Stop Centers, which provide free medical care, psychosocial support, and legal services to victims of IPV and child abuse, and short-term emergency shelter [21].

The National Policy against Gender-Based Violence shows that, despite the government's efforts in GBV prevention and response, there are still issues that need to be addressed, such as the persistence of some negative cultural beliefs and the victim's economic or livelihood reliance, which were found to be contributing factors to the rise in IPV [22]. Many people also believe gains in women's representation and protections reflect women's empowerment, which explains the dramatic increase in self-reported IPV against women [23]. Some other people assume that the COVID-19 pandemic, which impacted most of the sectors in the world, also played a role in the rise of intimate partner violence [24, 25].

The main goal of this study is to explore trends and correlates of intimate partner violence against women and men in Rwanda using two demographic health surveys (DHS 2015 and DHS 2020).

## Methodology

The data used in the study was extracted from the Rwanda Demographic and Health Surveys (RDHS) in 2014/2015 and 2019/2020. The data is based on nationally representative surveys of ever-married women aged 15–49 years and male aged 15–59 years conducted by the National Institute of Statistics of Rwanda (NISR) in collaboration with other international organizations (USAID, UN, CDC, UNICEF, UNFPA, and UNWOMEN) and government institutions such as the Ministry of Health and Rwanda Biomedical Center. The RDHS employed a two-stage sample design and was intended to provide estimates of key indicators at the national, urban, and rural levels, as well as for five provinces and each of Rwanda's 30 districts. The first stage involved selecting sample points (clusters) made up of delineated EAs (Enumeration Areas). The second stage involved systematic sampling of households. A household listing operation was carried out in each of the selected EAs, and households to be included in the survey were drawn at random from these lists. For this study, we restricted our sample to women (aged between 15 and 49 years) and men (aged between 15 and 59 years) who have been married or cohabiting in the past or are currently married or cohabiting. After the exclusion, the final samples used in the analysis were (for women: DHS2014/2015 = 8292, DHS2019/2020 = 8574, and for men: DHS2014/2015 = 3470, DHS2019/2020 = 3590).

### Outcome variable

In this study, the outcome variable was IPV. This variable was a man or woman's experience with at least one form of intimate partner violence (physical, sexual, or emotional). To derive physical violence, different questions were asked to participants, including "Did your husband or partner ever: push you, shake you or throw you something, slap you, kick you or drag you, strangle or burn you, threaten you with a knife/gun or other weapon?" Sexual violence was assessed by posing the questions: "Did your husband or partner ever physically force you into unwanted sex or to perform sexual acts you didn't want to?" Further, emotional violence was assessed by asking women if their partner "humiliated," "threatened to hurt or harm them," or "insulted or made them feel bad about themselves." Responses were then categorized as having ever experienced physical, sexual, or emotional violence and coded "1," while those who had never experienced any form of intimate partner violence were coded "0".

### Independent variables

The study included sociodemographic variables that are considered to be related to spousal violence. The ages of the women were included as a categorical variable with the following ranges: 15–24, 25–34, and 35–49 for women, and 15–24, 25–34, 35–49, and 50 and above for men. The wealth index was divided into five categories: poorest, poorer, middle, rich, and richest. The educational level of women and their husbands/partners was classified according to the highest level of education completed (no education level completed, primary education level, secondary education level, and higher education level), and women’s literacy (illiterate and literate). The variable of the province, which specified where women were located, was divided into five regions (south, west, north, east, and Kigali city). The type of residence was divided into two categories: urban and rural.

Some other variables used in this study were categorized as follows: household size (1–3, 4–5, and 6 and above), number of children aged five and below (none, 1 or 2, and 3 or 4), and working status (working and not working). Additionally, alcohol usage and respondents’ perceptions of people who make decisions about their own health care, earnings, purchases, and relatives’ visits were also used.

### Ethical consideration

The Rwanda National Institute of Statistics (NISR), Macro International Internal Review Board, and Rwanda National Ethics Committee all reviewed and approved the DHSs. The NISR granted us permission to use this deidentified data for this analysis.

### Statistical analysis

We used a Statistical Package for Social Science (SPSS), version 25, to perform all the analyses. Data were analyzed using frequencies and percentages to describe the demographic characteristics of respondents. The chi-square test was used to test the association between respondents’ sociodemographic variables and intimate partner violence. Furthermore, a multivariate logistic regression model was then performed to assess whether respondents’ social demographic characteristics influence their likelihood of experiencing intimate partner violence. For women: respondents’ age, education, place of residence, literacy, wealth index, involvement in decision making about their earnings, purchases, health and visits; partners’ age, education; number of children < 5 in household, number of household members were used as adjustment variables. For men: respondents’ age, education, frequency of being hit in last 12 months by other than wife/partner, number of wives or partners, and frequency of wife/partner being drunk were used as adjustment variables.

## Results

**Fig. 1:**
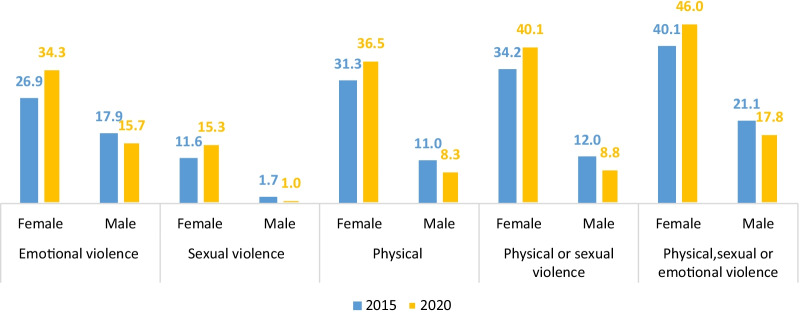
Trends in prevalence of IPV victimization

The results show that the prevalence of intimate partner violence (IPV) against women by their partners increased from 40.1% in 2015 to 46% in 2020 but declined from 21 to 17% for men in the same period. IPV was also found to have increased in all forms. Besides, the study found that physical violence was the most prevalent form for women, while emotional IPV was the most prevalent form for men (Fig. 1).

Of the total sample in 2015 and 2020, the majority of women and men were from rural areas, in the Southern and Eastern provinces, and aged 30 years and above. In terms of education, the majority of them had only a primary level (see Table 1).

**Table 1:** Selected sociodemographic characteristics of women used in study

	DHS2015		DHS2020	
	Women (N = 8292)	Men (N = 3470)	Women (N = 8574)	Men (N = 3590)
*Respondent's age*				
15–19	1.2	0.1	0.9	0.1
20–29	32.6	20.9	26.1	14.1
30–39	40.7	37.3	43.2	39.7
40–49	25.4	23.7	29.8	27.6
50 and above		17.9		18.5
*Partner's age*				
15–19	0.1	0.9	0.1	0.9
20–29	21.8	33.2	15.7	24.2
30–39	40.8	39.2	42.2	42.7
40–49	24.4	18.8	28.2	24.0
50–59	12.9	7.8	13.7	8.2
*Respondent's education*				
No education	17.3	16.3	13.8	14.3
Primary	69.1	70.7	65.4	69.0
Secondary	10.5	9.1	15.9	11.6
Higher	3.1	3.9	4.8	5.0
*Partner's education*				
No education	18.3		13.8	
Primary	67.3		66.7	
Secondary	10.4		13.5	
Higher	4.0		5.9	
*Respondent's literacy*				
Cannot read at all	24.1	21.1	19.9	19.9
Able to read only parts of sentence	8.5	8.2	10.2	9.8
Able to read whole sentence	67.4	70.7	69.9	70.3
*Respondent 's province*				
Kigali city	12.3	12.3	11.8	11.3
South	25.2	25.0	23.8	24.3
West	22.6	23.1	22.8	23.2
North	15.8	16.3	16.3	16.6
East	24.2	23.3	25.3	24.6
*Respondent's residence*				
Urban	22.7	21.9	21.6	20.3
Rural	77.3	78.1	78.4	79.7
*Respondent's wealth index*				
Poorest	21.4	17.1	22.4	19.5
Poorer	20.2	20.1	19.2	19.4
Middle	19.1	20.5	19.0	21.2
Richer	18.1	20.7	19.7	20.6
Richest	21.2	21.5	19.7	19.3
*Number of household members*				
1–3	23.6	24.4	22.5	22.4
4–5	40.9	38.7	43.3	41.8
6 and above	35.5	36.9	34.2	35.7
*Number of children 5 and under in household*				
None	29.1		28.7	
1 or 2	66.4		67.6	
3 or 4	4.4		3.7	
5 and above	0.1		0.1	

In bivariate analysis in 2015, most of the covariates were statistically associated with IPV for both women and men, for example, consumption of alcohol, level of education, literacy, wealth index, decision on personal health, decision on earnings, decision on household purchase, decision on visits to family or relatives. Other covariates associated with IPV in women included the woman's age and the level of education of her partner; in men, the number of wives or partners, the frequency of the wife/partner being drunk, and owning land alone or jointly were significantly associated with IPV (P value 0.05) (Table 2).

**Table 2:** Bivariate associations of IPV and socio-demographic characteristics

		DH2015				DH2020			
	Ever experienced IPV								
		Women		Men		Women		Men	
		No	Yes	No	Yes	No	Yes	No	Yes
*Respondent's age*	*P* value	0.002		0.054		0.035			0.363
15–19		0.9	1.7	0.1	0.0	1.7	0.6	0	0
20–29		39.0	30.9	22.9	18.1	29.3	26	16.4	11.8
30–39		40.0	44.1	39.9	43.7	45.0	48.2	43.0	45.7
40–49		20.0	23.3	23.1	19.1	24.4	25.3	26.0	27.3
50 and above		0.0	0	14.0	19.1	0	0	14.6	15.1
Total % (N)		100 (1139)	100 (764)	100 (1094)	100 (293)	100 (1052)	100 (895)	100 (1129)	100 (245)
*Partner's age*	*P* value		0.40		0.523		0.302		0.419
15–19		0.1	0.2	1.2	0.4	0.2	0	0.8	0.9
20–29		27.9	21.2	35.9	32.3	18.9	15.6	28.2	26.9
30–39		41.4	43.4	41.4	44.9	45.5	47.2	47.0	44.7
40–49		20.6	23.2	16.0	15.7	23.6	25.5	18.0	17.8
50 and above		10	12.1	5.5	6.7	11.8	11.7	6.0	9.6
Total % (N)		100 (1020)	100 (604)	100 (1080)	100 (254)	100 (967)	100 (699)	100 (1101)	100 (219)
*Respondent's education*	*P* value		0.000		0.008		0.000		0.058
No education		15.8	18.2	16.8	24.6	11	16.4	12.8	16.3
Primary		69.5	74.5	71.3	67.6	62.5	69.2	69.3	71.8
Secondary		11.5	6.8	7.9	5.8	20	13.1	12.9	9.8
Higher		3.2	0.5	4.0	2.0	6.5	1.3	5.0	2.0
Total % (N)		100 (1139)	100 (764)	100 (1094)	100 (293)	100 (1052)	100 (895)	100 (1129)	100 (245)
*Residence of respondent*	*P* value		0.264		0.425		0.005		0.658
Urban		22.8	17.5	22.3	20.1	19.9	17.9	20.0	18.8
Rural		77.2	82.5	77.7	79.9	80.1	82.1	80.0	81.2
Total % (N)		100 (1139)	100 (764)	100 (1094)	100 (293)	100 (1052)	100 (895)	100 (1129)	100 (245)
*Partner's education*	*P* value		0.000				0.000		
No Education		14.4	22.5			10.4	17.1		
Primary		70	69			68	72.5		
Secondary		11.4	7.2			14.3	8.7		
Higher		4.3	1.2			7.3	1.7		
Total % (N)		100 (1135)	100 (759)			100 (962)	100 (691)		
*Respondent's literacy*	*P* value		0.000		0.001		0.018		0.053
Cannot read at all		22.9	28.3	20.9	30.8	17	25	18.5	25.3
Able to read only parts of sentence		8.7	9.4	9.0	6.2	10.7	10.2	10.0	9.8
Able to read whole sentence		68.3	62.3	70.2	63.0	72.2	64.8	71.4	64.9
Total % (N)		100 (1134)	100 (763)	100 (1093)	100 (292)	100 (1052)	100 (893)	100 (1127)	100 (245)
*Respondent's Wealth index*	*P* value		0.000		0.001		0.000		0.111
Poorest		17	28.9	17.367	26.6	19.4	28.3	19.5	24.5
Poorer		21	20.8	21.389	21.5	19.2	22.8	20.1	20.4
Middle		20.5	19	20.750	22.9	18.5	18.2	23.0	26.1
Richer		18.6	17.9	21.115	16.0	22.2	17.7	20.3	14.7
Richest		22.9	13.4	19.378	13.0	20.6	13.1	17.1	14.3
Total % (N)		100 (1139)	100 (764)	100 (1094)	100 (293)	100 (1052)	100 (895)	100 (1129)	100 (245)
*Partner’s drink alcohol*	*P* value		0.000		0.000		0.000		0.000
No		14.2	10.7	67.1	44.7	23.2	20.6	71.8	49.0
Yes		85.8	89.3	32.9	55.3	76.8	79.4	28.2	51.0
Total % (N)		100 (1139)	100 (764)	100 (1094)	100 (293)	100 (1052)	100 (895)	100 (1126)	100 (245)
*Respondent currently working*	*P* value		0.162		0.657		0.026		0.916
No		43.6	21.5	0.5	0.3	50.6	20.7	1	1
Yes		56.4	78.5	99.5	99.7	49.4	79.3	99	99
Total % (N)		100 (1139)	100 (764)	100 (1094)	100 (293)	100 (1052)	100 (895)	100 (1129)	100 (245)
*Respondent's province*	*P* value		0.565		0.392		0.519		0.223
Kigali city		11.6	10.9	12.1	9.9	11	10.7	11.5	9.0
South		24.4	23.7	24.4	27.6	23.9	25.3	23.5	27.3
West		23.4	22.5	23.4	24.2	22.4	24.6	22.9	24.5
North		15.4	18.3	18.0	14.3	16.3	16.2	17.9	13.1
East		25.3	24.6	22.1	23.9	26.3	23.2	24.2	26.1
Total % (N)		100 (1139)	100 (764)	100 (1094)	100 (293)	100 (1052)	100 (895)	100 (1129)	100 (245)
*Respondent involved with decisions about her earnings*	*P* value		0.000		0.025		0.000		0.000
Respondent alone		12.4	24.8	18.5	25.6	17.5	29.1	13.0	22.5
Respondent and partner		76.9	57.5	79.6	71.2	75.1	58.3	84.6	70.5
partner alone		10.6	17.7	2.0	3.3	7.5	12.6	2.3	7.0
Total % (N)		100 (620)	100 (395)	100 (920)	100 (215)	100.1 (550)	100 (398)	100 (937)	100 (200)
*Respondent involved with decisions about her health*	*P* value		0.000		0.061		0.000		0.036
Respondent alone		16.9	27.2	42.4	51.6	21.9	36.4	46.0	52.5
Respondent and partner		67.4	54.5	53.8	45.7	61.3	42.6	51.0	42.5
Partner alone		15.7	18.4	3.7	2.8	16.8	20.9	3.0	5.0
Total % (N)		100 (1021)	100 (604)	100 (1079)	100 (254)	100 (966)	100 (697)	100 (1101)	100 (219)
*Respondent involved in decisions about household purchase*	*P* value		0.000		0.00		0.000		0.00
Respondent alone		7.4	14.8	27.1	37	4.8	10.9	23.5	39.3
Respondent and partner		69.3	50.6	69.7	56.7	76.9	59.1	70.1	53.4
Partner alone		23.2	34.7	3.2	6.3	18.3	30	6.4	6.8
Total % (N)		100 (1021)	100 (603)	100 (1079)	100 (254)	100 (966)	100 (697)	100 (1101)	100 (219)
*Respondent involved with decisions about her visits of friends or relatives*	*P* value		0.000				0.000		
Respondent alone		12.1	22.6			16.3	22.5		
Respondent and husband/partner		75.9	60.5			74.2	59.1		
Husband/partner alone		11.9	16.9			9.5	18.5		
Total % (N)		100 (101)	100 (603)			100 (963)	100 (699)		
*Household members*	*P* value		0.129		0.169		0.000		0.161
1–3		30.5	25.1	25.6	28.0	28.6	26.8	24.4	30.2
4–5		39.3	42.7	41.7	45.1	42.4	46.9	46.1	42.0
6 and above		30.2	32.2	32.7	27.0	29	26.3	29.6	27.8
Total % (N)		100 (1139)	100 (764)	100 (1094)	100 (293)	100 (1052)	100 (895)	100 (1129)	100 (245)
*Frequency of being hit in last 12 months by other than partner*					0.005				0.597
Not at all				90.4	81.2			90.8	87.8
Often				0.1	2.0			0.4	1.1
Sometimes				9.3	16.8			8.8	11.1
Total % (N)				100 (386)	100 (149)			100 (251)	100 (90)
*Number of wives or partners*	*P* value				0.002				0.15
One wife				97.315	93.3			96.9	95.0
More than one wife				2.685	6.7			3.1	5.0
Total % (N)				100 (1080)	100 (254)			100 (1101)	100 (219)
*Owns a house alone or jointly*					0.079				0.11
Does not own	*P* value			16.2	21.8			18.6	24.5
Alone only				34.6	33.8			28.0	28.6
Jointly only				48.6	44.4			48.7	44.1
Both alone and jointly				0.5	0.0			4.7	2.9
Total % (N)				100 (1094)	100 (293)			100 (1129)	100 (245)
*Owns land alone or jointly*					0.031				0.022
Does not own	*P* value			22.7	29.7			34.4	43.3
Alone only				28.6	28.7			25.4	25.7
Jointly only				48.3	40.6			36.1	29.0
Both alone and jointly				0.5	1.0			4.1	2.0
Total % (N)				100 (1094)	100 (293)			100 (1129)	100 (245)
Frequency of wife/partner being drunk					0.000				0.000
Never				81.9	41.9			61.8	26.4
Often	*P* value			1.1	10.0			0.3	9.6
Sometimes				17	48.1			37.9	64
Total % (N)				100 (359)	100 (160)			100 (317)	100 (125)

In 2020, many of the same covariates were also significantly associated with IPV against women, including age, level of education, literacy, wealth index, type of place of residence, and husband's consumption of alcohol, as well as involvement in decision-making about household purchase, earning, their health, and visits from friends and relatives. Furthermore, the frequency of the wife/partner being drunk, the husband being involved in household purchases, earnings, and health decisions, and the husband owning land alone or jointly were all associated with IPV against men (Table 2).

Multivariate logistic regression results are shown in Table 3. In 2015, the results revealed that women with an uneducated husband had a higher odd ratio of IPV (AOR = 5.570, CI 1.292–24.020), and women from the poorest households were more likely to experience IPV than women from rich households (AOR = 2.834, CI 1.637–4.908). Women whose husbands/partners drank alcohol were 3.021 times more likely to have IPV than women whose husbands did not drink alcohol (AOR = 3.021, CI 2.20–4.148), and women whose husbands were 30 to 39 years old were also associated with higher IPV (AOR = 2.797, CI 1.517–5.1158). Besides, women from families with 6 members or above were 1.68 times more likely to experience IPV compared to women from families with 1 to 3 members (AOR = 1.680, CI 1.043–2.706). Women involved in decisions about their own earnings (AOR = 0.576, CI 0.376–0.882), purchases (AOR = 0.472, CI 0.270–0.827) and versus their partner alone were protective factors. On the contrary, men’s findings showed that husbands of women who consume alcohol often (AOR = 13.354, CI 1.915–93.109) and sometimes (AOR = 3.842, CI 1.610–9.172) and those who have been hit sometimes in the last 12 months by someone other than their wife or partner (AOR = 5.498, CI 1.656–18.252) were more likely to experience IPV. Coming from a rich family was a protective factor against IPV against men (AOR = 0.21, CI 0.043–1.040).

**Table 3:** Multivariate analysis of association between IPV and socio-demographic characteristics

	2015		2020	
	AOR (95% CI)		AOR (95% CI)	
	Women	Men	Women	Men
*Respondent’s age*				
15–19	1.26 (0.27–5.84)	0.33 (0.08–1.38)	0.33 (0.06–1.83)	NA
20–29	0.60 (0.323–1.11)	1.46 (0.51–4.17)	1.05 (0.65–1.66)	NA
30–39	0.669 (0.40–1.11)	0.66 (0.21–2.12)	1.09 (0.75–1.59)	NA
40–49	Ref	0.71 (0.65–0.78)	Ref	NA
50–59	NA	Ref	NA	
*Partner's age*				
20–29	2.381 (1.168–4.856)*	NA	NA	NA
30–39	2.797 (1.517–5.158)*	NA	NA	NA
40–49	1.770 (1.014–3.089)*	NA	NA	NA
50 and above	Ref			
*Respondent's education*				
No education	2.38 (0.429–13.850)	0.37 (0.01–10.72)	1.16 (0.39–3.47)	2.57 (0.12–54.41)
Primary	2.91 (0.559–15.109	0.15 (0.01–3.28)	0.94 (0.36–2.41)	3.3 (0.24–45.66)
Secondary	3.11 (0.608–15.894)	0.05 (0.01–2.00)	0.97 (0.39–2.41)	5.12 (0.33–78.66)
Higher	Ref	Ref	Ref	Ref
*Partner’s education*				
No education	5.57 (1.29–24.02)*	NA	3.032 (1.117–8.24)*	NA
Primary	3.21 (0.77–13.24)	NA	2.459 (0.987–6.124)	NA
Secondary	2.56 (0.624–10.49)	NA	1.124 (0.444–2.843)	NA
Higher	Ref	Ref	Ref	
*Respondent's place of residence*				
Urban	1			
Rural	0.84 (0.55–1.29)	NA	NA	NA
*Respondent's literacy*				
Cannot read at all	1.00 (0.62–1.62)	1.28 (0.39–4.19)	1.02 (0.61–1.71)	1.19 (0.23–5.87)
Able to read only parts of sentence	0.98 (0.59–1.62)	0.15 (0.01–1.76)	0.86 (0.52–1.43)	0.45 (0.07–2.74)
Able to read whole sentence	1.00	1.00	1.00	1.00
*Respondent's wealth index*				
Poorest	2.83 (1.637–4.90)*	0.58 (0.11–3.13)	1.51 (0.87–2.63)	0.93 (0.18–4.78)
Poorer	1.89 (1.10–3.25)*	0.22 (0.04–1.19)	1.38 (0.79–2.41)	0.594 (0.11–3.21)
Middle	1.85 (1.08–3.17)*	0.47 (0.09–2.36)	1.07 (0.61–1.87)	1.522 (0.31–7.46)
Richer	1.66 (0.98–2.81)	0.21 (0.04–1.04)*	0.98 (0.57–1.66)	0.613 (0.11–3.49)
Richest	Ref	Ref	Ref	Ref
*Partner’s drink alcohol*				
No	Ref		Ref	
Yes	3.02 (2.20–4.14)*	NA	1.712 (1.126–2.606)*	NA
*Woman currently working*				
No	0.89 (0.49–1.60)	NA	0.864 (0.525–1.422)	NA
Yes	Ref		Ref	
*Respondent involved with decisions about her earnings*				
Respondent alone	Ref		Ref	
Respondent and husband/partner	0.57 (0.37–0.88)*		0.70 (0.46–1.09)	NA
Husband/partner alone	0.90 (0.51–1.57)		1.02 (0.56–1.86)	NA
*Respondent involved with decisions about her health*				
Respondent alone	Ref		Ref	
Respondent and husband/partner	0.77 (0.50–1.18)	NA	0.443 (0.304–0.644)*	NA
Husband/partner alone	0.49 (0.29–0.84)*	NA	0.56 (0.34–0.91)	NA
*Respondent involved with decisions about household purchase*				
Respondent alone	Ref		Ref	
Respondent and husband/partner	0.47 (0.27–0.82)*	NA	0.68 (0.36–1.30)	NA
Husband/partner alone	1.07 (0.59–1.93)	NA	0.94 (0.48–1.85)	NA
*Respondent involved with decisions about her visits*				
Respondent alone	Ref		Ref	
Respondent and husband/partner	1.03 (0.64–1.65)	NA	0.405 (0.21–0.748)*	NA
Husband/partner alone	0.93 (0.53–1.64)	NA	0.66 (0.38–1.12)	NA
*Number of children < 5 in household*				
None	Ref			
1 or 2	0.82 (0.57–1.17)	NA	NA	NA
3 or 4	1.04 (0.47–2.24)	NA	NA	NA
*Number of household members*				
1–3	Ref			
4–5	1.185 (0.81–1.74)	NA	NA	NA
6 and above	1.680 (1.04–2.71)*	NA	NA	NA
*Frequency of being hit in last 12 months by other than wife/partner*				
Not at all		Ref		Ref
Sometimes	NA	5.49 (1.65–18.25)*	NA	0.942 (0.236–3.756)
*Number of wives or partners*				
One		Ref		Ref
More than one wife	NA	0.83 (1.17–0.25)	NA	1.558 (0.303–8.003)
*Frequency of wife/partner being drunk*				
Never		Ref		Ref
Often	NA	13.30 (1.9–93.12)*	NA	10.27 (0.76–13.59)
Sometimes	NA	3.84 (1.61–9.17)*	NA	3.91 (1.55–9.87)*

AOR: Adjusted Odd Ratios; Ref: reference category; NA: Not Applicable: variables that are not applicable to men or women

*p ≤ 0.05

In multivariate analysis in 2020, women with uneducated husbands (AOR = 3.032, CI 1.117–8.235) and women whose husbands drank alcohol (AOR = 1.712, CI 1.126–2.606) were also associated with greater odds of IPV. Furthermore, women involved in decisions about their own health (AOR = 0.443, CI 0.304–0.644) or visiting their friends or relatives (AOR = 0.405, CI 0.21–0.748) were less likely to experience IPV. According to the men’s findings, men whose wives drank sometimes were likely to experience IPV (AOR: 3.914, CI 1.552–9.872). However, most of the sociodemographic characteristics were not statistically significant (Table 3).

## Discussion

To the best of our knowledge, this is the first study to examine the trends and correlates of IPV victimization in Rwanda using data from the Rwanda Demographic and Health Surveys 2014/2015 and 2019/2020. Overall, the study found that the prevalence of IPV against women increased from 40% in 2015 to 46% in 2020 and declined from 21 to 18% for men in the same period. With respect to the forms of IPV, physical violence was the most prevalent form of IPV among women, similar to other sub-Saharan countries [26], while the most popular form among men was emotional violence [27]. The increase in IPV among women could have been due to the greater empowerment and government policies toward gender equality that made women feel confident in reporting IPV [23]. Others argue that women’s empowerment increases a woman's risk of IPV because she is perceived to be challenging gender norms and threatening her male partner's dominant status or exercise of power [28–30]. We should also recognize the COVID-19 pandemic, which was identified as a major contributor in the growth of IPV by a number of researchers [24, 25, 31]. The prevalence rate of IPV in the current study is high when compared to the study conducted just before the pandemic [32]. The increase in prevalence of IPV was also reported in other countries in similar recent studies, for example, 42% in Malawi [33], 40% in Gambia [34], and 32.5% in Haiti [35], and 42.7%in Zimbabwe [36].

Similar to other research [15, 37], our study found that women were more likely to report intimate partner violence than men. There are numerous theories as to why men, in particular, may be hesitant to report domestic violence. In addition to their own feelings of shame, one reason could be a fear of not being believed and of being barred from contact with their children. There are also the reasons known by female victims of IPV: the desire for an intact family and the hope that their partner will change [15].

According to the study, the associated factors of intimate partner violence were women's wealth index, the husband/partner's age and level of education, partners' alcohol usage, and women’s participation in the decision making about their health, earnings, purchases, and visits to relatives or friends. The study revealed that women of low wealth were associated with a higher prevalence of intimate partner violence when compared to women of higher wealth. This could be due to wealth-driven empowerment, which eventually reduces their reliance on their partners [5, 26]. Previous research has also found that a woman's financial situation may be a protective factor against IPV [5, 26, 38, 39]. Poverty, on the other hand, has a high risk of exposing women to IPV because poor women rely heavily on their partners and may not be able to bargain [40].

The study discovered that uneducated women’s partners or husbands were more likely to commit violence to their intimate partners than higher educated husbands. These findings are similar to previous studies that reported that a partner's lower educational level increases the likelihood of IPV [41–43]. One possible explanation is that highly educated partners are more exposed to training and information, which cause them to believe and value the importance of respecting their partners' rights and freedoms. Their education also causes them to reject certain sociocultural practices that limit the value of women [34]. Education promotes compromising and caring behaviors as well as raises awareness of the negative consequences of violent behavior. As a result, the frequency of IPV decreases with the level of education of the husband.

The study also found that women whose husbands use alcohol are at high risk of experiencing intimate partner violence, which is consistent with findings from other studies [18, 23, 34, 35, 41, 42]. Other studies discovered that both men's and women's alcohol consumption was a risk factor for IPV [44]. Alcohol has a direct influence on cognitive and physical capacities, making individuals less self-controlling and less able to solve problems in relationships nonviolently [23].

Our findings indicated that women who were involved in decision-making about their own earnings, visiting relatives or friends, purchases, and health were less likely to experience IPV. This is also in accordance with the results obtained earlier [23, 45]. This can be explained in the context of women’s empowerment. Women’s involvement in household decision-making is one of the indicators of women’s empowerment [32], and when a woman is empowered, it can be an effective method of preventing her from IPV [28]. This is also supported by the initiative of promoting gender equality and empowerment, which is one of the United Nations' 17 Sustainable Development Goals [45].

### Strengths and limitations

This study has a number of notable strengths. The survey's strong methodologies and instrument ensured the validity and generalizability of our findings. Our findings may apply to the Rwandan married women and men since the data come from a nationally representative sample of ever-married women and men. However, given the cross-sectional nature of the data and its dependence on self-report, the results might be limited. Some men and women may have underreported violent abuse committed against them due to the conservative nature of many Rwandan societies, which could have led to an underestimation of the effects of those putative IPV risk or protective factors in the study.

## Conclusion

Rwanda has one of the highest self-reported rates of intimate partner violence against women in the world. The results indicated that women’s self-reported rates of IPV increased from 40% in 2015 to 46% in 2020. Therefore, IPV needs more assessment to know why it is continuously increasing so that it can be well managed. Since there is relationship between increase of IPV and education, the existing laws on domestic violence need to be known by the citizens, and there is a need for greater training in quality and quantity.

## Data Availability

The datasets used and/or analysed during the current study are available from the corresponding author on reasonable request.
